# Development of a diagnostic method for neosporosis in cattle using recombinant *Neospora caninum* proteins

**DOI:** 10.1186/1472-6750-12-19

**Published:** 2012-05-04

**Authors:** Jinhua Dong, Takahiro Otsuki, Tatsuya Kato, Enoch Y Park

**Affiliations:** 1 Laboratory of Biotechnology, Graduate School of Science and Technology, Shizuoka University, 836 Ohya, Suruga-ku, Shizuoka, 422-8529, Japan; 2 Laboratory of Biotechnology, Department of Applied Biological Chemistry, Faculty of Agriculture, Shizuoka University, 836 Ohya, Suruga-ku, Shizuoka, 422-8529, Japan

## Abstract

**Background:**

Neosporosis is an infectious disease primarily of cattle and dogs, caused by intracellular parasite, *Neospora caninum*. Neosporosis appears to be a major cause of abortion in dairy cattle worldwide and causes to huge economic loss to dairy industry.

**Results:**

Recombinant surface associated antigen 1 (NcSAG1), NcSAG1 related sequence 2 (NcSRS2) and the dense granule antigen 2 (NcGRA2) of *N. caninum* were expressed either in silkworm or in *Escherichia coli* and purified. The purified recombinant proteins bound to the *N. caninum*-specific antibodies in serum samples from infected cattle as revealed by an enzyme-linked immunosorbent assay (ELISA). By co-immobilizing these recombinant proteins, a novel indirect ELISA was developed for detection of neosporosis. With the use of 32 serum samples, comprising 12 positive serum samples and 20 negative serum samples, the sensitivity and specificity of the assay were found to be 91.7 and 100%, respectively. Seventy-two serum samples from dairy farms were also tested and one was diagnosed with neosporasis with both this method and a commercial assay.

**Conclusions:**

A diagnostic method employing recombinant proteins of *N. caninum* was developed. The method showed high sensitivity and specificity. Diagnostic test with field serum samples suggested its applicability to the practical diagnosis of neosporosis.

## Background

Neosporosis is an infectious disease primarily of cattle and dogs, caused by *Neospora caninum*. *N. caninum* is an obligate intracellular protozoan parasite, which was first recognized in dogs in Norway [1], and has been found to infect a wide variety of mammals such as cattle, sheep, goats, deer and horses [2-4]. Owing to the similarity of *N. caninum* to *Toxoplasma gondii*, neosporosis was misdiagnosed as *T. gondii* infection for many years [5]. Dogs are the definitive host of *N. caninum* and cattle are usually its intermediate host. Neosporosis now appears to a major cause of abortion in dairy cattle worldwide and causes to huge economic loss to dairy industry [6]. Most studies of *N. caninum* have been focused on infections in dairy cattle [7].

*N. caninum* was identified by immunohistochemistry in two aborted fetuses from Argentina in 1998 [8]. There are several developmental stages of the parasite, which differ in size and distribution. The rapidly dividing tachyzoite stage is found within many different cells of the host. Tissue cysts are found primarily in nervous tissue and the oocyst stage is in feces excreted from definitive hosts of the parasite. The main mechanism of infection is due either the reactivation of latent tissue cysts or result from the ingestion of oocysts during the gestation period. Currently, there is no effective method of control or medical treatment of neosporosis, except the use of intensive farm management practices to reduce the likelihood of infection.

*N. caninum* possesses specialized secretory organelles called rhoptries, micronemes, and dense granules. Proteins secreted from these organelles are considered to play an essential role in intracellular parasitism by this protozoan [9]. Dense granule antigens (NcGRAs) of *N. caninum* are major components of both the vacuoles surrounding tachyzoites and the cyst wall that surround slower-growing bradyzoites [10], and therefore NcGRAs might be promising diagnostic tools and important protective antigens. Proteins displayed on the surfaces of intracellular pathogens are believed to play critical roles in infection. The *N. caninum* surface associate antigen 1 (NcSAG1) and NcSAG1 related sequence 2 (NcSRS2) have been identified as major surface antigen proteins of *N. caninum* tachyzoites, and were demonstrated to be immune-dominant and involved in interactions between the tachyzoite and the host cell [11]. Their predominant antigenicity was also demonstrated by their recognition by antisera from *Neospora*-infected animals [12].

Various diagnostic methods for neosporosis have been developed. For instance, the indirect fluorescent antibody test (IFAT) was employed to detect anti-*N. caninum* antibodies in sera of cattle, to evaluate the infection status [13]. Besides IFAT, other serological diagnostic tools such as immunoblotting [14], agglutination tests [15] and enzyme-linked immunosorbent assays (ELISA) [16-18] are also available. For serological evaluation of neosporosis, total proteins of the parasite or recombinant antigens are generally used. Recombinant antigens are easily produced in large quantities and can be standardized readily.

With the aims of achieving a reliable diagnosis and developing vaccines, many proteins of *N. caninum* have been studied. However, the number of recombinant proteins that have been investigated as vaccine candidates is limited. The surface protein NcSRS2, expressed in recombinant vaccinia virus, offered adequate protection against transplacental passage and was found to limit parasite dissemination [19]. Other proteins, such as NcSAG1 [20] and NcMIC3 [21] were also reported to have high antigenicity. A number of proteins from *N. caninum* have been expressed as inclusion bodies in *E. coli*, but proteins refolded *in vitro* may not have the complete original structure, resulting in limited antigenicity.

In this paper, we report the expression and purification of recombinant *N. caninum* proteins, NcGRA2, NcSRS2, and NcSAG1, as soluble proteins in *E. coli* or silkworms. Furthermore, a diagnostic method for neosporosis was developed using the recombinant proteins.

## Results

### Expression of MBP-NcGRA2, MBP-NcSRS2 and NcSAG1, and purification

**Figure 1:**
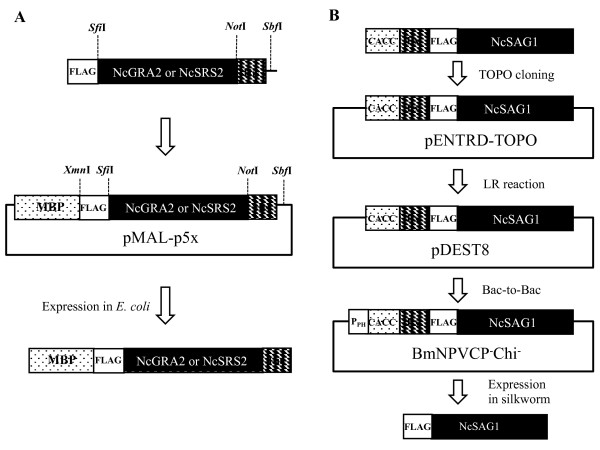
**Panels A and B show genetic construction for NcGRA2 and NcSRS2, and NcSAG1, respectively.** MBP: maltose binding protein; P_PH_: polyhedrin promoter; Bx: bombyxin signal peptide.

**Figure 2:**
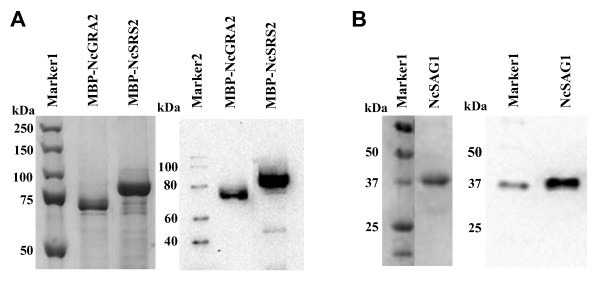
**Expression of recombinant *Neospora caninum* proteins.** MBP-NcGRA2 and MBP-NcSRS2 were expressed in *E. coli* and purified (**A**). NcSAG1 was expressed in silkworms and purified (**B**). Left and right sides of each panel show SDS-PAGE and western blot, respectively. MBP-NcGRA2 and -SRS2 were detected with an anti-His tag antibody, NcSAG1 with an anti-FLAG antibody. Markers 1, 2, and 3 denote Precision Plus Protein^TM^ Dual Colors Standards, MagicMark XP Western Protein Standards, and Precision Plus Protein™ WesternC™ Standards, respectively.

The genes for NcGRA2 and NcSRS2 were amplified by polymerase chain reaction (PCR) using appropriate primers (Table 1) and cloned into a pMAL system, with which recombinant proteins could be expressed as fusion proteins with Maltose Binding Protein (MBP), as described in Figure 1A. MBP-NcGRA2 and MBP-NcSRS2 were expressed as soluble forms in *E. coli*. The periplasmic fraction and culture supernatant were collected and purified with Talon Co^2+^-immobilized resin. The purified proteins were separated by sodium dodecyl sulfate–polyacrylamide gel electrophoresis (SDS-PAGE) and blotted onto polyvinylidene fluoride (PVDF) membranes for western blot. The molecular weight of the fusion protein MBP-NcGRA2 was estimated at approximately 64 kDa (Figure 2A), which was in agreement with that deduced from its amino acid sequence. The other purified protein, MBP-NcSRS2, with an estimated molecular weight of 90 kDa, was also confirmed (Figure 2A). NcSAG1 gene was cloned into a Bacmid system and expressed in silkworms (Figure 1B). Recombinant NcSAG1 was purified as a single band at about 38 kDa (Figure 2B), which matched its estimated molecular weight.

**Table 1:** Primers used in this study

**Primer name**	**Sequence (5’– 3’)**
GRA2SfiNcoFor	ACGGCCCAGCCGGCCATGGCCGATTTTTCT
GRA2NotHisBack	TCTGCGGCCGCATTGACTTCAGCTTCT
GRA2NotHisFor	AGTCAATGCGGCCGCGGTCGAGCACCACCA
SbfI-His4Back	ACATCCTGCAGGTCAGTGGTGGTGGTG
FLAGSfiFor	GACTACAAGGATGACGATGACAAGGCGGCCCAGCCGGCCA
SRS2SfiNcoFor	CGGCCCAGCCGGCCATGGCGCCGTTCAAGT
SRS2NotBack	TCTGCGGCCGCGGGGGAATCGCCGTTCTCT
CACC-bx-FLAG-HRV3C Forward	CACCATGAAGATACTCCTTGCTATTGCATTAATGTTGTCAA CAGTAATGTGGGTGTCAACAGACTACAAGGATGACGATGAC AAGGGTGCACTTGAAGTCCTCTTTCAG
SAG1Forword	TATGGTACCGATCAGAAAAATCACCTCTA
SAG1Reverse	ATAGAGCTCTCACGCGACGCCAGCCGCTAT

### Antigenicity of recombinant *Neospora caninum* proteins and optimization of assay

**Figure 3:**
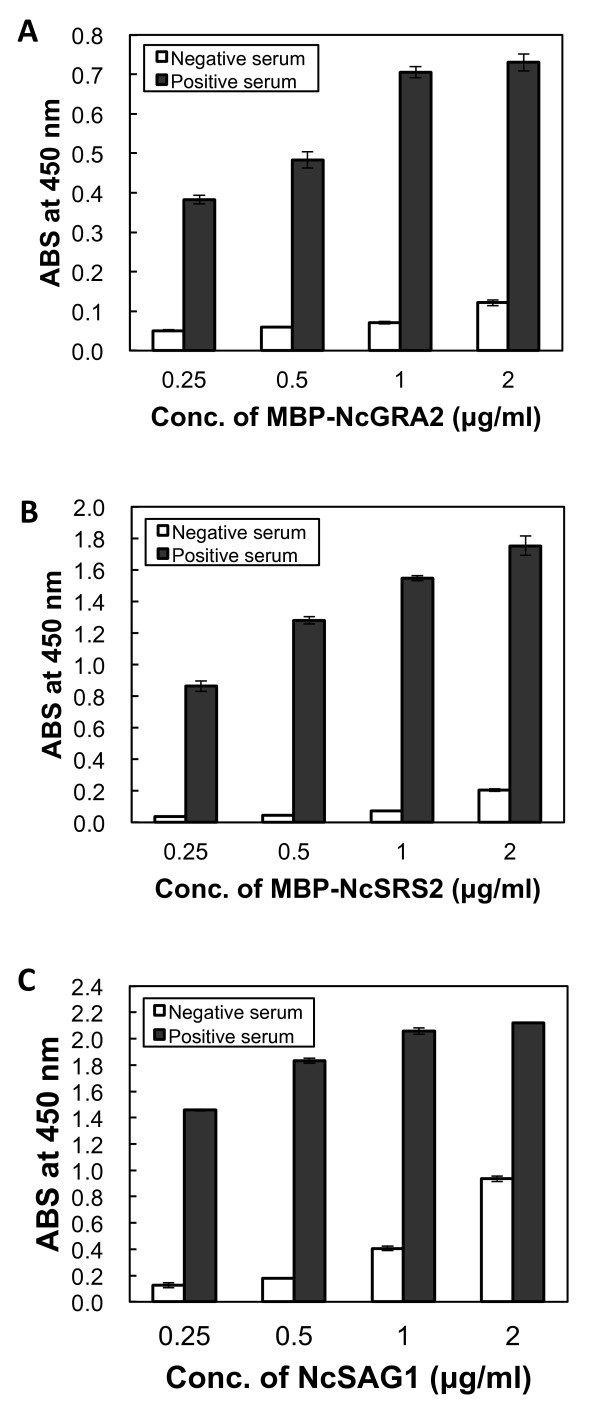
**Optimization of recombinant *Neospora caninum* protein amounts for the diagnostic assay.** MBP-NcGRA2 (**A**), MBP-NcSRS2 (**B**), or NcSAG1(**C**) was immobilized on a 96-well microplate at concentrations of 0.25, 1, 0.5, and 2 μg/ml. ELISAs were performed to test the anti-*N. caninum* antibodies in negative and positive cattle serum samples (n = 3).

To check the antigenicity of the expressed *N. caninum* proteins and to optimize the amount of protein used for immobilization, an indirect ELISA was performed. Purified MBP-NcGRA2, MBP-NcSRS2, or NcSAG1 was diluted to final concentrations of 0.25, 0.5, 1.0, and 2.0 μg/ml, and immobilized on a microplate at 4°C overnight, respectively. After blocking, neosporosis-negative or -positive cattle sera were added, followed by anti-bovine antibody HRP, and detected by the addition of 3,3',5,5'-tetramethylbenzidine (TMBZ) substrate. High signal intensity was observed in the wells to which serum from neosporosis-positive cattle had been added (Figure 3). On the other hand, only a low signal was detected for neosporosis-negative samples. This demonstrates that the recombinant *N. caninum* proteins retained the properties of the native proteins of *N. caninum*, and that MBP-NcGRA2, MBP-NcSRS2, and NcSAG1 have antigenicity sufficient for immunization.

To optimize the amounts of proteins used for immobilization, each recombinant protein at 0.25, 0.5, 1.0, and 2.0 μg/ml was analyzed. Based on the results, the percent positive value (PP value), which was defined as the ratio of the mean absorbance value of sample serum to that of the positive control, as a percentage, was calculated for each concentration of protein. Concentrations at which the lowest PP values were obtained were considered to be most suitable. For MBP-NcGRA2, the PP values were 13.2, 12.5, 10.0, and 16.6 at 0.25, 0.5, 1.0, and 2.0 μg/ml, respectively, suggesting that MBP-NcGRA2 worked well at 0.25–2 μg/ml and the most suitable concentration should be around 1.0 μg/ml. For MBP-NcSRS2, the PP values were 4.3, 3.5, 4.7, and 11.7. MBP-NcSRS2 worked soundly within the range of 0.25 μg/ml to 2 μg/ml, and 0.5 μg/ml gave the lowest PP value. PP values for NcSAG1 at 0.25, 0.5, 1.0, and 2.0 μg/ml were 8.6, 9.7, 19.7, and 44.0. NcSAG1 at a lower concentration of 0.25–1 μg/ml is appropriate for the detection of anti–*N. caninum* antibodies in cattle serum.

We also optimized the protein concentrations for co-immobilization by a similar method, as described above. A protein cocktail solution including MBP-NcGRA2, MBP-NcSRS2, and NcSAG1 at concentrations of 1.0, 0.5, and 0.25 μg/ml, respectively, gave an ideal result, and was adopted as the detector for neosporosis.

### Evaluation of *Neospora Caninum* detection

An indirect ELISA method was designed to detect bovine *N. caninum*-specific antibodies in serum with the recombinant *N. caninum* protein cocktail described above. Anti-*Neospora* antibodies–recombinant *N. caninum* proteins was formed a complex with HRP-conjugated anti-bovine antibodies. The result was observed visually, and the optical density at a wavelength of 450 nm was measured using a microplate reader.

**Figure 4:**
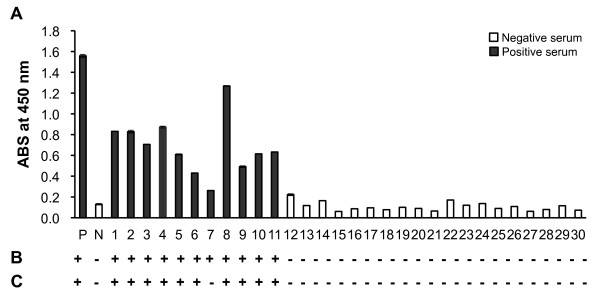
**Evaluation of cattle serum samples with an ELISA.** MBP-NcGRA2 (1 μg/ml), MBP-NcSRS2 (0.5 μg/ml), and NcSAG1 (0.25 μg/ml) were co-immobilized on a 96-well microplate. Anti-bovine IgG-HRP was used as the secondary antibody. (**A**) The ELISA results with the new method developed in this study (n = 3). (**B**) Diagnostic results with a commercial ELISA kit and © those with this method “+”and “–” represent positive and negative results, respectively.

Thirty-two serum samples, comprising 12 positive and 20 negative sera, were employed to evaluate the *N. caninum* detector. The samples were also tested with individual of MBP-NcGRA2, MBP-NcSRS2, NcSAG1, or a commercial *N. caninum* isocom ELISA kit (SVANOVA Biotech AB, Boehringer Ingelheim Svanova, Uppsala, Sweden). If the PP value was smaller than 20, the cattle were determined to be negative; if it was equal to or greater than 20, the cattle were thought to be infected with *N. caninum*. As shown in Figure 4 and Table 2, among the 12 neosporosis-positive samples, 11 were distinguished and only no.7 serum sample was misclassified. None of the 20 neosporosis-negative serum samples was misclassified with the cocktail detector. On the basis of these data, the sensitivity of this assay was found to be 91.7% and the specificity 100%, when compared with the commercial *N. caninum* iscom ELISA kit.

**Table 2:** Percent positivity values of test serum samples

	Protein for immobilization^*^				**Neospora caninum iscomELISA kit**
**Sample number**	**MBP-** **NcGRA2**	**MBP-** **NcSRS2**	**NcSAG1**	**Co- immobilization**	
Positive	100	100	100	100	100
Negative	10.0	3.5	10.1	8.3	5.3
1	56.5	48.3	51.5	53.4	27.6
2	47.3	52.5	58.2	53.2	30.0
3	57.0	43.2	22.4	45.3	27.6
4	59.3	37.8	51.0	56.0	39.0
5	36.0	29.5	53.1	39.1	41.7
6	19.0	21.2	28.8	27.6	35.9
7	18.5	13.0	14.8	16.8	20.5
8	92.1	73.0	78.3	81.4	56.6
9	45.2	22.5	30.2	31.5	26.8
10	45.6	42.1	37.8	39.5	50.1
11	53.2	36.8	27.2	40.6	38.4
12	15.3	12.3	10.2	14.3	4.1
13	10.0	3.4	5.3	7.6	6.2
14	12.3	4.8	11.8	10.6	17.5
15	4.1	5.7	2.4	3.9	6.3
16	7.3	4.2	5.6	5.6	10.3
17	4.3	5.0	8.3	6.2	2.3
18	5.6	4.1	5.2	5.0	3.0
19	5.3	4.8	7.2	6.5	5.3
20	7.1	3.7	4.4	5.8	3.2
21	3.2	2.5	6.5	4.2	2.1
22	13.3	9.0	8.9	11.0	2.4
23	9.5	6.4	8.2	7.8	2.9
24	6.6	10.2	8.1	8.8	2.1
25	4.5	11.4	4.7	5.8	3.3
26	5.1	8.3	6.0	7.0	2.7
27	5.2	4.7	3.8	4.0	3.0
28	8.0	4.3	4.8	5.1	3.4
29	9.3	5.9	7.3	7.4	3.6
30	6.2	3.6	4.2	4.7	4.7

^*^The concentrations of MBP-NcGRA2, MBP-NcSRS2 and NcSAG1 were 1.0, 0.5, and 0.25 μg/ml for both individual and co-immobilization.

ELISAs were also carried out with single protein immobilized for the 32 serum samples. When we only immobilized MBP-NcGRA2, two serum samples, no. 6 and no. 7 serum samples were misclassified, giving a sensitivity of 83.3% (Table 2). Similar result to protein cocktail was obtained when only either MBP-NcSRS2 or NcSAG1 was immobilized, however, PP values for most serum samples were lower than immobilization of protein cocktail. Co-immobilization of three proteins improved the reliability of the assay with a single protein.

### Diagnosis of neosporosis with serum samples from dairy cattle

**Figure 5:**
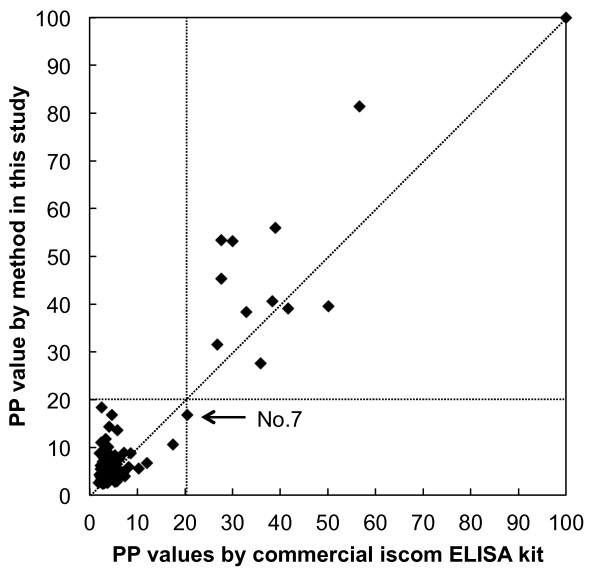
**Comparison of percent positivity values between the new method and a iscom ELISA kit.** The percent positivity values in Table 2 and Additional file  1 were plotted. Serum samples with value less than 20 are considered negative, those with higher value than 20, positive.

Another group, comprising 72 serum samples from dairy farms in the Shizuoka Prefecture of Japan, was also tested with both the method developed in this study and the *N. caninum* iscom ELISA kit. The diagnostic results matched very well, and one serum sample was found to be positive for infection with *N. caninum* using both methods. An additional file shows this in more detail (see Additional file  1). The PP values of all the serum samples tested by the method developed in this study and by a commercial iscom ELISA kit were compared. The new method showed high values in the positive area (PP value > 20) in most positive samples (Figure 5).

## Discussion

Neosporosis is a very common disease worldwide, and losses to livestock farms that result from neosporosis-related abortions are a huge problem. Given that no therapy or effective vaccine is available currently, diagnosis and protection are very important to dairy farms. In this study, we developed a diagnostic method based on recombinant *N. caninum* proteins, MBP-NcGRA2, MBP-NcSRS2, and NcSAG1. An ELISA with co-immobilization of those three proteins gave ideal results in both the development and validation steps. We also tried to immobilize those proteins separately; however, the results were not as good as with co-immobilization. For instance, one more positive sample was misclassified when we immobilized only MBP-NcGRA2 for use in the ELISA. Individual immobilization of MBP-NcSRS2 or NcSAG1 resulted in lower PP values for positive serum samples than co-immobilization of three proteins.

Other proteins, such as NcMIC1 [22], NcMIC3 [21], and GRA7 [23] have also been reported to have antigenicity. Therefore addition of those proteins to the diagnostic test might increase the sensitivity of the assay. In addition, some assays use inactivated total proteins of the parasite as a detector for the diagnosis of neosporosis. However, given that some proteins of *N. caninum* have high similarity to those of *T. gondii*[5], total proteins of parasites might result in misclassification. NcGRA2, NcSRS2 and NcSAG1 have identities of 33%, 43% and 51% to those of *T. gondii*, respectively. According to the report of Nishikawa et al. [24], no cross-reaction was observed between NcSRS2 and that of *T. gondii*. Therefore, they could be used to distinguish these two infectious diseases.

Two proteins were expressed in an *E. coli* protein expression system, which is a cost-effective method of producing large quantities of high-quality recombinant proteins [25]. However, this approach is often insufficient for soluble expression of recombinant proteins. The Structural Genomics Center has estimated that up to 50% of all prokaryotic proteins are insoluble when expressed in *E. coli*[26]. So far, many proteins of *N. caninum* have been expressed in *E. coli*, and most of them were expressed as inclusion bodies [27]. In this case, refolding procedures are necessary in order to obtain biofunctional proteins. Although some proteins recover their activity after refolding treatments, it is still difficult to reproduce the complete properties of the native protein.

We expressed soluble NcGRA2 and NcSRS2 by fusing them with MBP, which is capable of functioning as a general molecular chaperone in the context of a fusion protein [28]. Moreover, MBP did not hinder the immunoassay used in this study, which suggests that it may aid the production of protein on a large scale. We also tried to express NcSAG1, but unfortunately we could not obtain a soluble fraction. However, use of the BmNPV bacmid expression system, with both the cysteine-protease and chitinase genes deleted (BmNPV-*CP*^−^*Chi*^−^), led to successful expression of NcSAG1 in silkworm larvae as a soluble fraction, with less protein degradation. The viral protease and chitinase activities in the hemolymph of the silkworm larvae were reduced by 95 and 50%, respectively [29].

Given that the tachyzoite is a very infectious stage of *N. caninum*, many studies of vaccination and detection have been focused on this stage. NcGRA2 is usually contained in dense granules, and it is highly expressed in culture-derived tachyzoites, therefore it has been studied as a potential vaccine candidate. However, so far there has been no study on the diagnosis of neosporosis that has used this protein as a detector. In this study, this protein bound to *N. caninum*-specific antibodies in serum samples from cattle, which suggests that NcGRA2 is a good candidate protein for use in diagnostic assays. The combination of NcGRA2, NcSRS2, and NcSAG1 resulted in reliable detection of neosporosis.

The new method showed high values in the positive area in most positive samples than a commercial iscom ELISA kit, which suggests that this method may show a higher level of response to anti *Neospora* antibodies than the commercial ELISA kit. Among the 104 samples tested, only one positive sample, no.7 serum sample, was misclassified as negative using our method ( Additional file 1 and Figure 4). However, the PP value of this sample calculated with the commercial ELISA kit was 20.5, which was very close to the boundary value for interpretation; that obtained with our method was 16.8.

In addition to cattle, naturally occurring neonatal or fetal infections caused by *Neospora*-like protozoa have been described in dogs, goats [30], horses [31,32], and sheep [3]. Therefore, this diagnostic method may have wide applicability to other animal species.

## Conclusions

A diagnostic method employing recombinant proteins of *N. caninum* was developed. The method showed high sensitivity and specificity, suggesting its applicability to the practical diagnosis of neosporosis.

## Methods

### Materials

Genomic DNA of *N. caninum* NC-1 strain (ATCC no.50843) was purchased from ATCC (USA). The plasmid pMAL-p5x was obtained from New England Biolabs (NEB; Ipswich, MA, USA). The *E*. *coli* strains used in this study were DH5α (Agilent Technologies, La Jolla, CA, USA) for general cloning and BL21 (DE3) (Novagen, Madison, WI, USA) for protein expression. The plasmids pENTR/DTOPO and pDEST8 were purchased from Invitrogen (San Diego, CA, USA). Restriction and modification enzymes were purchased from Takara-Bio (Shiga, Japan), Toyobo (Osaka, Japan), Roche Diagnostics (Tokyo, Japan) or NEB. Oligonucleotides were synthesized either by Operon (Tokyo, Japan) or Invitrogen (Tokyo, Japan). Other chemicals, reagents and antibodies, unless otherwise indicated, were obtained from Sigma (St Louis, MO, USA) or Wako Pure Chem. (Osaka, Japan). Cattle serum samples for the diagnosis of neosporosis were provided by Shizuoka Prefecture Tobu Livestock Disease Diagnostic Center (101 Nitta Kannamicho Tagata–gun, Shizuoka Prefecture, Japan). The commercial *N. caninum* iscom ELISA kit, used as a reference, was purchased from SVANOVA Biotech AB (SVANOVA Biotech AB, Boehringer Ingelheim Svanova, Uppsala, Sweden).

### Construction of pMAL-p5x-NcGRA2 and pMAL-p5x-NcSRS2

The NcGRA2gene without its original signal peptide was amplified by polymerase chain reaction (PCR) using the primer pair GRA2SfiNcoFor/GRA2NotHisBack from the plasmid pET-NcGRA2, which was constructed previously by our group. His tag DNA was amplified from a pET52b(+) plasmid with primers GRA2NotHisFor and SbfI-His4Back. The NcGRA2 and His tag genes were linked by splicing overlap PCR. FLAG tag was attached to the NcGRA2 gene by PCR with the primers FLAGSfiFor and SbfI-His4Back. The amplified DNA was purified with an Illustra^TM^ GFX^TM^ PCR DNA and Gel Band Purification kit (GE Healthcare), followed by digestion with *Sbf*I and cloning into a predigested pMAL-p5x vector with *Xmn*I/*Sbf*I to construct the expression plasmid pMAL-p5x-NcGRA2. The NcSRS2 gene without the signal peptide gene was amplified from plasmid pET-NcSRS2 with primers SRS2SfiNcoFor and SRS2NotBack, followed by purification and digestion with *Sfi*I/*Not*I. It was cloned into *Sfi*I/*Not*I-digested pMAL-p5x-NcGRA2 to construct pMAL-p5x-NcSRS2 (Figure 1A). The sequences of the primers used in this study are shown in Table 1. *E. coli* DH5α was transformed with pMAL-p5x-NcGRA2 or pMAL-p5x-NcSRS2, and cultured to yield plasmid DNA. The sequences of the genes inserted in the expression vectors were confirmed on a CEQ 8000 sequencer (Beckman Coulter, Carlsbad, CA, USA).

### Construction of rBmNPV-NcSAG1 bacmid

The full-length NcSAG1 gene was amplified from pET-SAG1 by PCR using SAG1Forward and SAG1Reverse primers (Table 1). For TOPO cloning and expression in silkworms, CACC, bombyxin (bx) signal peptide, the FLAG tag sequence and a protease cleavage site, HRV3C, were attached by PCR using primers CACC-bx-FLAG-HRV3CForward and SAG1Reverse. The amplified gene was inserted into pENTR/D-TOPO (Invitrogen) by the topoisomerase reaction. Using this plasmid, the full-length NcSAG1 gene was inserted into pDEST8 using Gateway technology (Invitrogen) to construct pDEST-NcSAG1, which was then used to transform *E. coli* BmDH10Bac *CP*^−^*Chi*^−^[29]. Blue–white selection was performed to identify colonies that contained the recombinant bacmid. Recombinant *Bombyx mori* nucleopolyhedrovirus (rBmNPV-NcSAG1) bacmids were extracted from white transformants and insertion of the NcSAG1 gene was confirmed by PCR.

### Expression of MBP-NcGRA2 and MBP-NcSRS2, and purification

*E. coli* BL21(DE3) cells were transformed with pMAL-p5x-NcGRA2 or pMAL-p5x-NcSRS2, grown on Luria–Bertani (LB) medium agar plates containing 100 μg/ml ampicillin (LBA) at 37°C for 16 h. A single colony was cultured in LBA medium at 37°C with shaking at 200 rpm overnight, and inoculated subsequently into 1 liter of LBA medium. When the *E. coli* cells had multiplied to 2–4 × 10^8^ cells/ml (A_600_ ~0.5), 0.3 mM of isopropyl-β-d-galactopyranoside (IPTG) was added, and the culture was incubated further at 30°C for 8 h. The *E. coli* cells were harvested by centrifugation at 4000 × g for 20 min at 4°C. The periplasmic fraction was extracted according to the protocol provided by NEB. Supernatant of culture was also collected and precipitated by adding ammonium sulphate at a final concentration of 75% (w/w). His-tagged MBP-NcGRA2 and MBP-NcSRS2 were purified from the periplasmic fraction and concentrated supernatant with Talon Co^2+^-immobilized resin (Clontech, Takara-Bio) according to the instructions provided by the manufacturer. The purified proteins were analyzed by SDS–PAGE and western blot.

### Expression of NcSAG1 in silkworms and purification

NcSAG1 was expressed in silkworms. The recombinant bacmid containing the NcSAG1 gene (10 μg) was mixed with one-tenth volume of DMRIE-C (Invitrogen) and incubated at room temperature for over 45 min. Fifty microliters of this mixture was injected into a silkworm larva on the first day of the fifth instar larvae (Ehime Sansyu Co. Ltd., Ehime, Japan). Injected silkworm larvae were reared for 5–7 days, and the hemolymph collected was centrifuged to remove hemocytes at 2400 × g for 10 min at 4°C. The supernatant was used as a hemolymph sample for purification. NcSAG1 was purified with an ANTI-FLAG M2 Affinity Gel (Sigma) according to the instructions provided by the manufacturer.

### SDS-PAGE and western blot

The expressed MBP-NcGRA2, MBP-NcSRS2, and NcSAG1 were analyzed by SDS–PAGE as described by Laemmli [33], and by western blot. Ten microliters of the protein samples were separated by SDS-PAGE and blotted onto a PVDF (Bio-Rad). After blocking the membrane with phosphate-buffered saline (PBS: KH_2_PO_4_, 1.47 mM; Na_2_HPO_4_, 8.10 mM; NaCl, 136.89 mM; KCl, 2.68 mM) containing 2% skimmed milk (MPBS) at room temperature for 2 h, 1 μg/ml of anti-His antibody (for detection of MBP-NcGRA2 and MBP-NcSRS2) or anti-FLAG antibody (for NcSAG1) was added to the membrane in an appropriate volume. After incubation for 1 h, the membrane was washed with PBST (PBS containing 0.1% Tween 20) three times, and anti-mouse IgG HRP conjugate (Promega, Madison, WI) was poured on prior to incubation for 1 h. After washing with PBST three times, the bands were developed with ECL Plus reagents (GE Healthcare) and detected with a VersaDoc Imaging System (Bio-Rad).

### Indirect ELISA

An indirect ELISA was performed to investigate the antigenicity of recombinant proteins and diagnose neosporosis. Each well of a microplate was coated with 100 μl/well of recombinant protein in PBS overnight at 4°C. After blocking with 200 μl of MPBS, 100 μl of serum samples from *N. caninum*-infected cattle or healthy cattle, diluted 1000-fold in MPBS, were added and the plates incubated for 1 h at 25°C. Each well was washed three times with PBST, and incubated with 100 μl/well of 5000-fold diluted HRP/anti-bovine antibody conjugate in MPBS. Anti-*N. caninum* antibodies bound to recombinant proteins were detected by the addition of TMBZ solution.

### Optimization of diagnostic assay

To optimize the immobilization of recombinant *N. caninum* proteins, series diluted proteins were immobilized for indirect ELISA. Based on the results of the ELISA, the PP value was calculated for each concentration of protein. After the optimum concentrations of MBP-NcGRA2, MBP-NcSRS2, and NcSAG1 had been decided, a cocktail solution containing all three kinds of protein at the appropriate concentration was made to act as the *N. caninum* detector.

A sample group, comprising 12 positive sera and 20 negative sera, was employed to evaluate the detector. The positive and negative control samples used for optimization of the protein concentration were also included among the 32 serum samples. By immobilization of MBP-NcGRA2, MBP-NcSRS2, NcSAG1, separately, or the protein cocktail, ELISAs were carried out and the PP value for each sample was calculated. The sensitivity and specificity of the assay were calculated according to the diagnostic results.

### Application of diagnostic assay to serum samples from dairy cattle

Seventy-two serum samples from cattle, collected from dairy farms in the Shizuoka Prefecture of Japan, were also tested with both the method developed in this study and a commercial iscom ELISA kit for the diagnosis of *N. caninum*. The PP values were calculated for each samples with both methods and compared by plotting the values.

## Competing interests

The authors declare that they have no competing interests.

## Authors' contributions

JD carried out the expression and purification of MBP-NcGRA2, MBP-NcSRS2, and the immunoassays, conceived of the study, participated in its design and drafted the manuscript. TO carried out the expression and purification of NcSAG1 and immunoassays. TK participated in the design of the study. EYP conceived of the study, participated in its design, and drafted the manuscript. All authors read and approved the final manuscript.

## Supplementary Material

Additional file 1 Percent positivity values of field serum samples. Click here for file
