# Supplementary material for: Development of a diagnostic method for neosporosis in cattle using recombinant Neospora caninum proteins
Source: BMC Biotechnol. 2012 May 4;12:19. doi: 10.1186/1472-6750-12-19 (PMC3441611; doi:10.1186/1472-6750-12-19)
Supplement: Additional file 1 — Percent positivity values of field serum samples. [file 1472-6750-12-19-S1.doc]

**Additional file**

Supplementary Table. Percent positivity values of field serum samples

| Sample number | Co-immobilization of recombinant NC proteins | Neospora caninum iscom ELISA kit |
| --- | --- | --- |
| 31 | 16.8 | 4.6 |
| 32 | 18.4 | 2.5 |
| 33 | 8.8 | 8.6 |
| 34 | 7.2 | 3.4 |
| 35 | 6.8 | 3.6 |
| 36 | 3.0 | 2.5 |
| 37 | 3.8 | 2.4 |
| 38 | 8 | 5.1 |
| 39 | 3.2 | 4.3 |
| 40 | 3.3 | 3.9 |
| 41 | 8.2 | 4.5 |
| 42 | 4.8 | 2.9 |
| 43 | 3.9 | 3.3 |
| 44 | 7.3 | 3.7 |
| 45 | 8.9 | 7.2 |
| 46 | 5.8 | 2.6 |
| 47 | 2.9 | 5.7 |
| 48 | 4.5 | 2.6 |
| 49 | 4.7 | 3.3 |
| 50 | 10.1 | 3.9 |
| 51 | 2.5 | 3.0 |
| 52 | 8.8 | 3.0 |
| 53 | 6.1 | 3.7 |
| 54 | 2.7 | 3.7 |
| 55 | 10.5 | 3.0 |
| 56 | 2.6 | 1.9 |
| 57 | 3.7 | 3.8 |
| 58 | 11.8 | 3.4 |
| 59 | 6.3 | 3.5 |
| 60 | 2.4 | 2.7 |
| 61 | 4.3 | 2.1 |
| 62 | 7.2 | 4.0 |
| 63 | 4.5 | 3.3 |
| 64 | 6.7 | 12.0 |
| 65 | 5.1 | 5.1 |
| 66 | 4.0 | 7.4 |
| 67 | 6.2 | 4.1 |
| 68 | 9.8 | 3.4 |
| 69 | 6.4 | 5.4 |
| 70 | 5.5 | 2.3 |
| 71 | 2.8 | 5.3 |
| 72 | 3.9 | 2.3 |
| 73 | 3.5 | 3.4 |
| 74 | 3.9 | 3.6 |
| 75 | 5.3 | 3.4 |
| 76 | 2.7 | 3.0 |
| 77 | 3.3 | 4.1 |
| 78 | 38.4 | 32.9 |
| 79 | 3.6 | 2.8 |
| 80 | 4.1 | 5.4 |
| 81 | 7.4 | 5.8 |
| 82 | 5.9 | 8.2 |
| 83 | 3.8 | 4.2 |
| 84 | 4.3 | 2.6 |
| 85 | 7.8 | 5.3 |
| 86 | 2.9 | 3.1 |
| 87 | 5.6 | 5.7 |
| 88 | 3.5 | 3.3 |
| 89 | 6.2 | 3.3 |
| 90 | 8.7 | 3.4 |
| 91 | 2.6 | 3.7 |
| 92 | 7.5 | 4.3 |
| 93 | 3.0 | 2.8 |
| 94 | 4.5 | 7.0 |
| 95 | 2.8 | 2.3 |
| 96 | 3.2 | 2.4 |
| 97 | 5.8 | 5.7 |
| 98 | 2.7 | 3.9 |
| 99 | 5.2 | 3.0 |
| 100 | 13.6 | 5.8 |
| 101 | 2.9 | 2.9 |
| 102 | 4.9 | 7.2 |
